# Evaluation of the Indazole Analogs of 5-MeO-DMT and Related Tryptamines as Serotonin Receptor 2 Agonists

**DOI:** 10.1021/acsmedchemlett.3c00566

**Published:** 2024-01-19

**Authors:** Navoda Jayakodiarachchi, Mallory A. Maurer, Daniel C. Schultz, Cayden J. Dodd, Analisa Thompson Gray, Hyekyung P. Cho, Olivier Boutaud, Carrie K. Jones, Craig W. Lindsley, Aaron M. Bender

**Affiliations:** Warren Center for Neuroscience Drug Discovery and Department of Pharmacology, Vanderbilt University, Nashville, Tennessee 37232, United States

**Keywords:** Serotonin, psychedelic, tryptamine, indazole, SAR

## Abstract

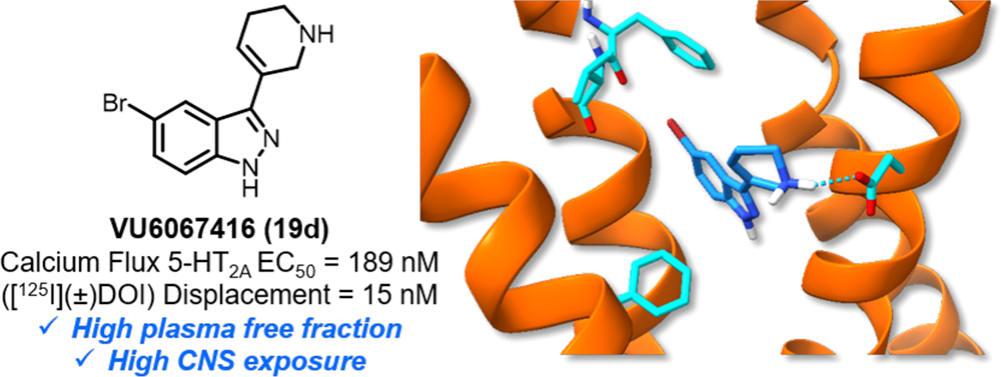

Herein, we report the synthesis and characterization of a novel set of substituted indazole-ethanamines and indazole-tetrahydropyridines as potent serotonin receptor subtype 2 (5-HT_2_) agonists. Specifically, we examine the 5-HT_2_ pharmacology of the direct indazole analogs of 5-methoxy-*N*,*N*-dimethyltryptamine (5-MeO-DMT) and related serotonergic tryptamines, and highlight the need for rigorous characterization of 5-HT_2_ subtype selectivity for these analogs, particularly for the 5-HT_2B_ receptor subtype. Within this series, the potent analog VU6067416 (**19d**) was optimized to have suitable preclinical pharmacokinetic properties for *in vivo* dosing, although potent 5-HT_2B_ agonist activity precluded further characterization for this series. Additionally, *in silico* docking studies suggest that the high potency of **19d** may be a consequence of a halogen-bonding interaction with Phe234^5.38^ in the 5-HT_2A_ orthosteric pocket.

*N*,*N*-Dimethyltryptamine (DMT) and 5-methoxy-*N*,*N*-dimethyltryptamine (5-MeO-DMT) are among the most powerful canonical agonists for the serotonin receptor 2A (5-HT_2A_) and are known to produce profound changes in perception and mood after systemic dosing.^1−3^ These compounds, along with many other classical psychedelics, have recently seen a resurgence in clinical profiling for a number of indications including depression, post-traumatic stress disorder (PTSD), obsessive-compulsive disorder (OCD), cluster headaches, end of life anxiety, and many others.^4−6^ Indeed, a number of encouraging clinical reports, highlighting the efficacy of these and related psychedelics, have begun to emerge in the literature, and as of 2021 at least 70 registered clinical studies using psychedelics have been reported.^4^ Results of a phase 3 clinical study evaluating 3,4-methylenedioxymethamphetamine (MDMA), a phenethylamine in the entactogen class of psychedelics,^7^ were recently published and demonstrated that the compound was a safe and effective treatment for severe symptoms observed in PTSD patients.^8^

In addition to DMT and 5-MeO-DMT, the tryptamine class of psychedelics includes psilocybin (a phosphate ester prodrug of the active 5-HT_2A_ agonist psilocin)^9^ and LSD (a semisynthetic ergoline alkaloid),^10^ and the first specific associations of tryptamine structures with psychedelic experiences were first described in the literature at least as early as the 1940s.^6,11,12^ Given the long history of this class of compounds and the number of recent reports detailing various tryptamine analogs as novel 5-HT_2A_ agonists,^13−17^ it is therefore perhaps surprising that very few reports exist of a direct indazole analog of a serotonergic-type tryptamine. The first such report, published in 1957, describes a synthetic method by which to access the direct 1*H*-indazole analog of tryptamine itself (**3**, see Figure 1), although no pharmacology data are reported.^18^ In keeping with the recent resurgence in psychedelic research, this manuscript remained one of the only reports describing an indazole-substituted serotonergic until very recently, when a selection of patent applications emerged in the literature which describe a wide variety of *N*-substituted tryptamines and tryptamine isosteres^19,20^ (including the direct 1*H*-indazole analog of 5-MeO-DMT).^20^

**Figure 1:**
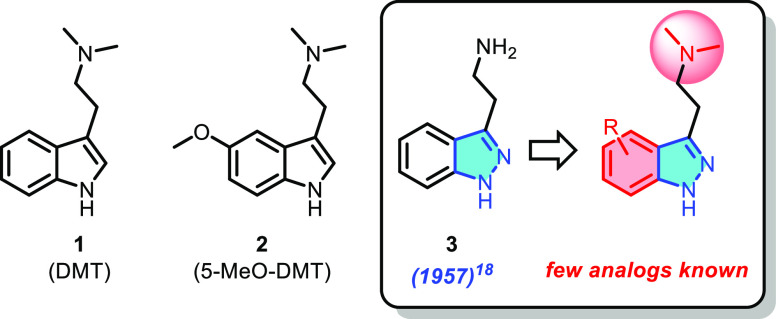
Chemical structures of DMT, 5-MeO-DMT, and 1*H*-indazole **3**.

Incorporation of the indazole motif has yielded compounds with excellent properties across a number of drug discovery programs^21−25^ and is present in no less than 43 compounds undergoing clinical evaluation (as of 2021).^26^ Additionally, indazoles are known to act as effective bioisosteres for indoles and phenols, and are often superior with respect to plasma clearance, oral bioavailability, and metabolic stability.^27−29^ We were therefore also interested in examining the indazole isosteres of classical psychedelics (including 5-MeO-DMT) to ultimately (1) profile the 5-HT_2A_ potency and serotonin-subtype selectivity of 2-*aza*-5-MeO-DMT and compare our data to the known literature (with a parallel emphasis on the generation of novel analogs) and (2) understand the overall properties of an indazole series of tryptamines with respect to preclinical pharmacokinetics (PK).

The direct 1*H*-indazole analog of 5-MeO-DMT, compound **6a**, was accessed as shown in Scheme 1. Tertiary amides **5a** and **5b** were generated from commercially available methyl ester **4** via hydrolysis and amide coupling, followed by reduction with either lithium aluminum hydride or lithium aluminum deuteride to generate amines **6a**–**6c**. 1-Methylindazole analog **11** was synthesized as shown in Scheme 2. Briefly, triple alkylation of carboxylic acid **7**, followed by ester reduction, oxidation, and reductive amination gave **11** (attempts to generate 1*H*-indazoles **6a**–**6c** using a similar sequence were unsuccessful owing to the apparent instability of indazole aldehydes similar to **10** but lacking the 1-methyl substitution). 5-Chloro- and 5-hydroxyindazoles (**14** and **16**, respectively) were generated using a similar amide reduction sequence to **6a**–**6c**, as shown in Scheme 3. All analogs were then assessed for functional potency across all 5-HT_2_ subtypes (Table 1).

**Scheme 1:**
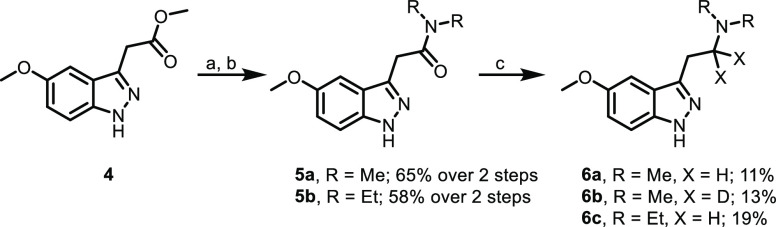
Synthesis of Indazoles **6a**–**6c** (a) LiOH, THF, H_2_O, rt; (b) dimethylamine hydrochloride or diethylamine, HATU, DIPEA, DMF, rt; (c) LiAlH_4_, or LiAlD_4,_ THF, rt.

**Scheme 2:**
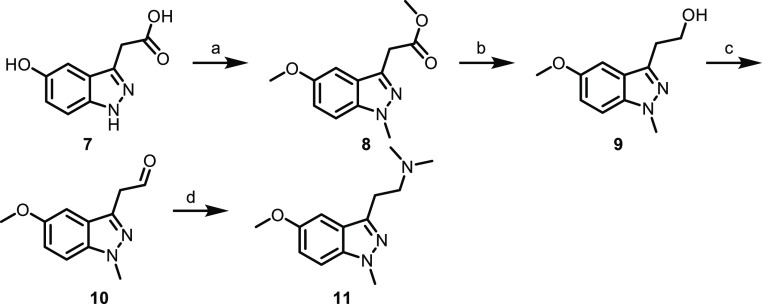
Synthesis of indazole **11** (a) MeI, Cs_2_CO_3_, DMF, rt, 27%; (b) DIBAL, DCM, −78 °C to rt, 67%; (c) Dess–Martin periodinane, DCM, rt, 91%; (d) dimethylamine hydrochloride, NaBH(OAc)_3_, DCM, rt, 33%.

**Scheme 3:**
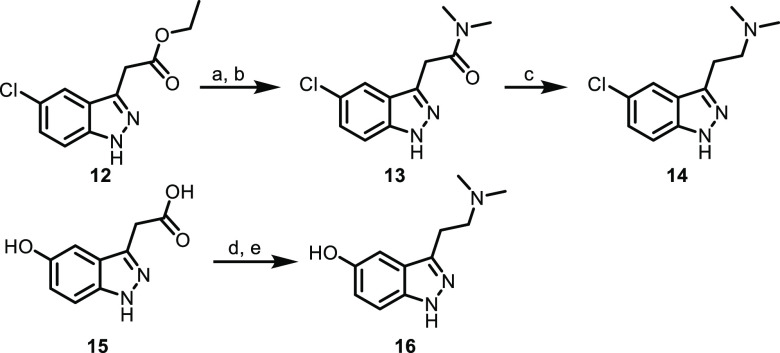
Synthesis of Indazoles **14** and **16** (a) LiOH, THF, H_2_O, rt.; (b) dimethylamine hydrochloride, HATU, DIPEA, DMF, rt, 26% over 2 steps; (c) LiAlH_4_, THF, rt, 14%; (d) dimethylamine hydrochloride, HATU, DIPEA, THF, DMF, rt; (e) LiAlH_4_, THF, rt, 11% over 2 steps.

**Table 1:**
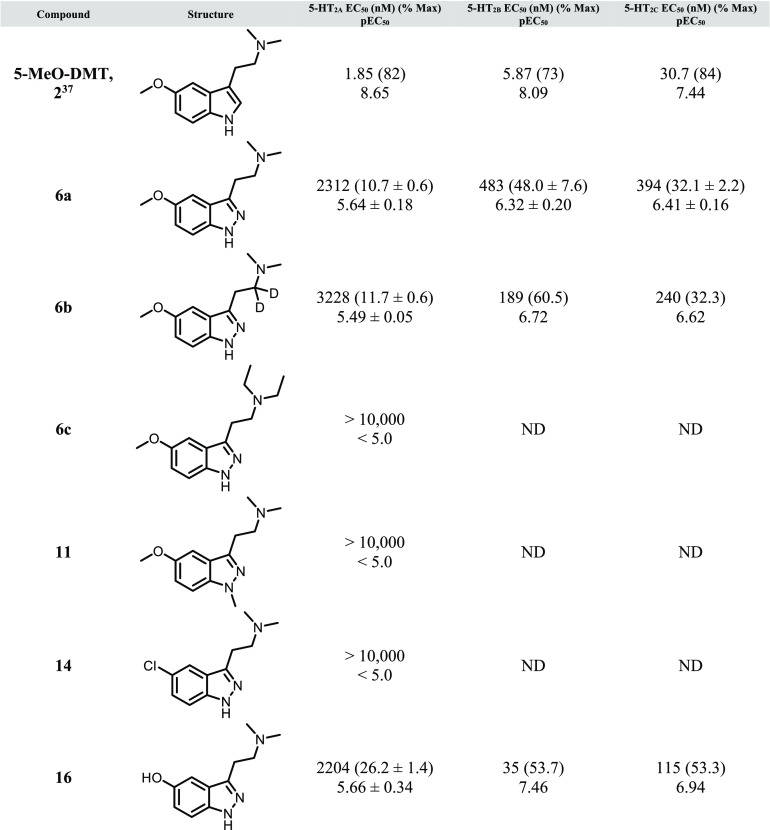
*In Vitro* Functional Potency for Compounds **2**, **6a**–**c**, **11**, **14**, and **16**^a^

a Calcium mobilization assays using human 5-HT_2A_-CHO, 5-HT_2B_-HEK293, and 5-HT_2C_-CHO cells. Data represent (*n* = 1 to 3) independent experiments performed in duplicate (data are ±SEM). See Supporting Information for additional details. ND = not determined.

In our hands, compound **6a** (the direct 1*H*-indazole analog of 5-MeO-DMT) was found to have low micromolar activity for 5-HT_2A_, with higher potency at 5-HT_2B_ and 5-HT_2C_ (and was less potent than the indole parent compound across all 5-HT_2_ subtypes). 1-Methyl analog **11** was markedly less potent at 5-HT_2A_ compared to both 5-MeO-DMT and **6a**. g*em*-Deutero analog **6b** was approximately equipotent at 5-HT_2A_ relative to its proteo-counterpart **6a**. (Recently, this type of deuterium incorporation was found to increase the *in vitro* stability for a series of DMT analogs in human hepatocyctes.^16^ In the case of the present indazole analogs, **6b** was found to have only marginally lower predicted clearance in human hepatic microsomes compared to **6a** (human CL_hep_ = 11.7 and 12.1 (mL/min)/kg, respectively), although a more complete metabolic picture (the effect of deuterium on MAO-mediated oxidation, etc.) would likely be obtained using hepatocytes.) Diethylamine **6c** and 5-chloroindazole **14** displayed no appreciable 5-HT_2A_ functional activity up to 10 μM, whereas 5-hydroxy analog **16** displayed similar potency to **6a** for 5-HT_2A_ (with higher potency for the other subtypes). Within this set, the relatively higher potency observed for **6a** and **16** align with the available published data for the corresponding tryptamines, in which a 5-MeO or 5-OH substitution in the context of the *N*,*N*-dimethylamine motif (5-MeO-DMT and bufotenin, respectively) are among the most potent tryptamines described in the literature.^30,31^ Because the more potent analogs in the present series do not show appreciable selectivity for 5-HT_2A_ relative to 5-HT_2B_ and 5-HT_2C_, and in fact are largely 5-HT_2B_-preferring, this appreciable 5-HT_2B_ agonist activity may elicit problematic cardiotoxicities for these and related tryptamines.^32^ Historically, there are no examples of orthosteric tryptamines with high selectivity across 5-HT_2_ subtypes due to the highly conserved nature of the orthosteric binding pocket, although examples of substituted phenethylamines with higher 5-HT_2A_ selectivity have been reported.^33−35^ Recently, additional chemotypes with some degree of 5-HT_2A_ subtype selectivity have started to emerge in the literature, and an allosteric approach may prove fruitful toward this end.^19,20,36^

Interestingly, the direct 1*H*-indazole analog of 5-MeO-DMT, compound **6a**, was previously described to be moderately potent for 5-HT_2A_ (5-HT_2A_ EC_50_ = 203 nM, *E*_max_ = 70%), with high selectivity relative to 5-HT_2B_ (EC_50_ > 10 μM) and, to a lesser extent, 5-HT_2C_ (EC_50_ = 532 nM, *E*_max_ = 72%).^20^ This large discrepancy between reports for the 5-HT_2B_ subtype selectivity profile for this compound is noteworthy (483 nM in our hands vs >10 μM), given that agonist activity (nor-dexfenfluramine and related clinical 5-HT_2B_ agonists) at this receptor presents a well-validated risk for cardiotoxicity.^32^ Although it is possible that differences in cell line background and/or receptor expression levels may partially account for this disparate selectivity data (see Supporting Information), this finding highlights the need for rigorous characterization of 5-HT_2A_ agonists (both known and novel) across multiple cell backgrounds and functional readouts if such compounds are to be safely profiled in the clinic.

Recently, Kaplan and colleagues utilized an ultralarge virtual docking approach to discover, among other novel 5-HT_2A_ chemical scaffolds, a series of *aza*-tryptamines in which the ethanamine pendant is cyclized to give a tetrahydropyridine moiety (see Table 2 for representative example **(*R*)-69**).^38^ Interested in examining this type of modification in the context of our indazole series, we synthesized tetrahydropyridine-indazoles **19a**–**f** and **21** as shown in Scheme 4. Briefly, commercially available substituted indazoles **17a**–**f** were coupled to *tert*-butyl 5-(4,4,5,5-tetramethyl-1,3,2-dioxaborolan-2-yl)-3,6-dihydropyridine-1(2*H*)-carboxylate using standard Suzuki–Miyaura chemistry, followed by Boc-deprotection to give compounds **19a**–**f**. Piperidine **20** was synthesized from **18a** via olefin hydrogenation prior to Boc-deprotection, and 5-phenyl analog **21** was synthesized via Suzuki–Miyaura coupling/Boc-deprotection from **18d**. Functional potency across all 5-HT_2_ subtypes is summarized in Table 2.

**Scheme 4:**
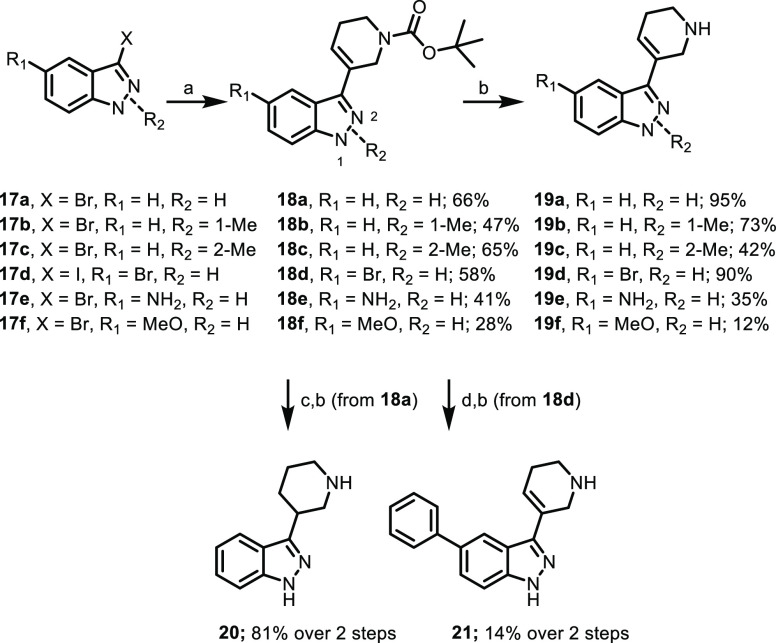
Synthesis of Indazoles **19a**-**f**, **20**, and **21** (a) *tert*-Butyl 5-(4,4,5,5-tetramethyl-1,3,2-dioxaborolan-2-yl)-3,6-dihydropyridine-1(2*H*)-carboxylate, K_2_CO_3_, PdCl_2_(dppf)·DCM, 1,4-dioxane, H_2_O, 110 °C; (b) HCl, DCM, rt; (c) 10% Pd/C, ammonium formate, MeOH, 60 °C; (d) phenylboronic acid, K_2_CO_3_, PdCl_2_(dppf)·DCM, 1,4-dioxane, H_2_O, 110 °C.

**Table 2:**
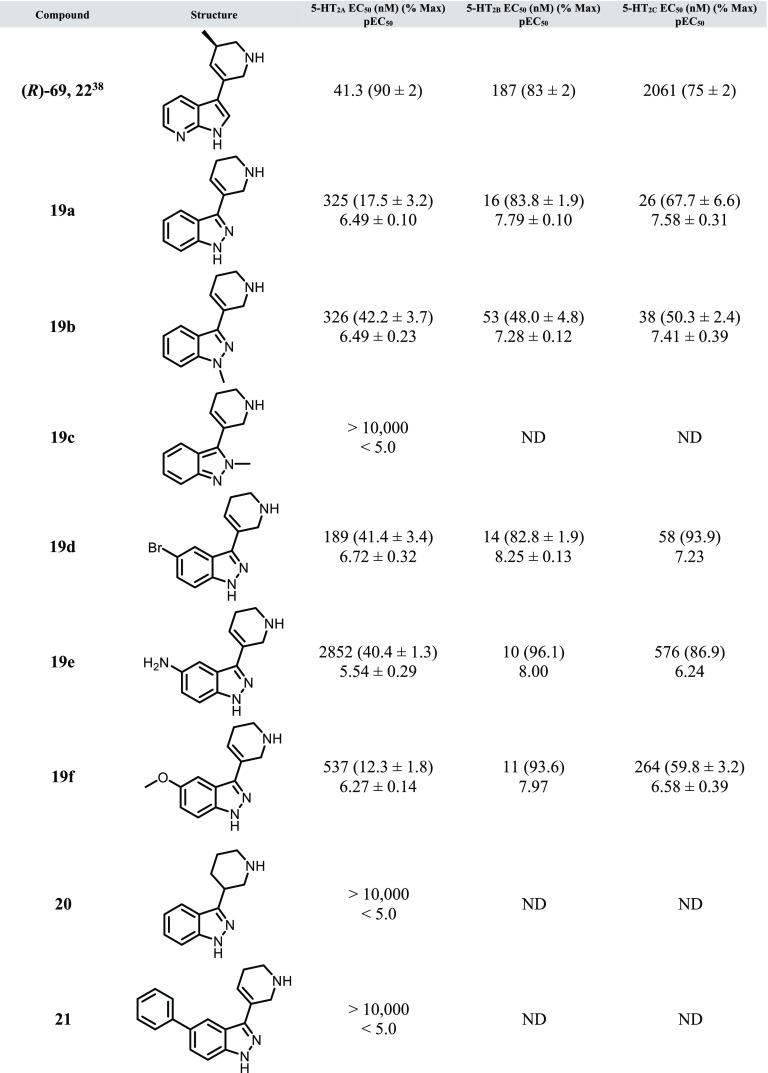
*In Vitro* Functional Potency for Compounds **19a**–**f** and **20**–**22**^a^

a Calcium mobilization assays using human 5-HT_2A_-CHO, 5-HT_2B_-HEK293, and 5-HT_2C_-CHO cells. Data represent (*n* = 1 to 3) independent experiments performed in duplicate (data are ±SEM). See Supporting Information for additional details. ND = not determined.

In contrast to the acyclic series (Table 1), 1*H*-indazole **19a** and its 1-methyl counterpart **19b** were found to be equipotent with respect to 5-HT_2A_, with both displaying approximately 10-fold higher agonist potency for 5-HT_2B_ and 5-HT_2C_ (2-methylindazole **19c** was found to be inactive at 5-HT_2A_ up to 10 μM, indicating the importance of the spatial arrangement of the methyl group on the indazole scaffold). All substitutions examined at the 5-position, with the exception of 5-phenyl analog **21**, were found to be tolerated (**19d**–**f**), with 5-bromo analog **19d** in particular showing high agonist potency across all 5-HT_2_ subtypes (189 nM for 5-HT_2A_). Ring reduction to give saturated piperidine analog **20** was not tolerated, indicating the necessity of the olefin constraint for 5-HT_2A_ activity. During the preparation of this manuscript, a related patent application containing a series of tetrahydropyridine-linked indoles and indazoles was disclosed, in which additional SAR around this type of 5-HT_2A_ scaffold is detailed.^19^ Within this report, the structures and functional 5-HT_2A_ potency data for **19a** and **19f** are reported, and interestingly, each compound appears significantly more potent compared to our findings (5-HT_2A_ EC_50_ values of 9.9 and 17.8 nM, respectively), although no selectivity relative to 5-HT_2B_ and 5-HT_2C_ is reported for these analogs. In our hands, **19a** and **19f** appear to be strongly 5-HT_2B_-preferring relative to 5-HT_2A_, indicating that the selectivity for 5-HT_2A_ in the context of the tetrahydropyridine series may prove challenging. As with the acyclic analogs described in Table 1, this potent 5-HT_2B_ agonist activity is concerning with respect to the potential to induce pulmonary arterial hypertension (PAH), valvular heart disease (VHD), and related cardiopathies.^32^ A functional agonist profile at 5-HT_2B_, however, does not necessarily guarantee cardiotoxicity, and in fact partial agonists for this receptor have been shown to prevent and treat Sugen-hypoxia-induced PAH in mice.^39^ Furthermore, signaling bias may play a role in determining the cardiotoxic potential of a given compound; compounds including ropinirole and BW723C86 are not known to induce cardiotoxicity despite being potent functional agonists in the Ca^2+^ calcium flux assay.^40,41^ Further characterization of the present compounds will be needed in order to fully understand any associated risks.^32^

Encouraged by the high potency of 5-bromo analog **19d** (VU6067416), we examined this analog in a battery of *in vitro* and *in vivo* pharmacokinetic (PK) assays. VU6067416 (**19d**) was found to have low predicted hepatic clearance (CL_hep_) in human microsomes, with higher turnovers observed for rodent species. Additionally, **19d** displayed a high fraction unbound (*f*_u_) in plasma across species, as well as a low predicted P-gp efflux, indicating high potential for brain penetration in human. In a rat iv PK study utilizing cassette dosing, **19d** showed moderate plasma clearance (CL_p_) and high *V*_ss_, with a 2.8 h half-life and a high total brain to plasma ratio (*K*_p_) of 5.4. These parameters, which are summarized in Table 3, are encouraging with respect to the high potential for brain exposure and free drug across species.

**Table 3:** *In Vitro* and *in Vivo* PK Parameters for VU6067416 (**19d**)^a^

parameter	value
*in vitro*	
CL_hep_ ((mL/min)/kg)	5.6 (human); 58 (rat); 57 (mouse)
*f*_u_	0.12 (human); 0.13 (rat); 0.12 (mouse)
P-gp efflux ratio (*P*_appA-B_ (10^–6^ cm/s))	1.3 (7.9)
rat PK cassette (iv, 0.2 mg/kg)	
CL_p_ ((mL/min)/kg)	34.2
*V*_ss_ (L/kg)	6.33
*t*_1/2_ (h)	2.8
*K*_p_ (measured at 0.25 h)	5.4

a See Supporting Information for additional experimental information.

**19d** was found to fully displace radiolabeled racemic 2,5-dimethoxy-4-iodomethamphetamine ([^125^I](±)DOI) in a competition binding experiment for the 5-HT_2A_ receptor (**19d** IC_50_ = 15 nM),^42^ suggesting an orthosteric binding profile. Previous literature has demonstrated the potential for halogenated 5-HT_2A_R substrates to form a halogen bond with either or both of the backbone carbonyls of Phe234^5.38^ and Val235^5.39^ in the 5-HT_2A_ orthosteric pocket.^43^ To explore this possibility in the context of the present series, VU6067416 (**19d)** was docked to an active state of 5-HT_2A_R (PDB code 7RAN)^38^ using AutoDock VinaXB (Figure 2A).^44^ While traditional 5-HT_2A_R agonist binding interactions with Phe340^6.52^ (π–π with indazole) and Asp155^3.32^ (salt bridge with amine) are retained, the distance between the bromine and Phe234^5.38^ backbone carbonyl oxygen is slightly outside the typical cutoff for halogen bond formation between a bromine and a carbonyl oxygen (3.74 vs 3.37 Å),^45^ rendering the occurrence of this phenomenon uncertain in this context. Given the potency of **19d** relative to other compounds in the present series and the limitations of rigid docking, though, it is possible that a halogen bond does form. Furthermore, an overlay of the cryo-EM bound pose of 5-HT_2A_R agonist **(*R*)-69**(38) with the docked pose of **19d** indicates a high degree of similarity between the two conformations, lending credence to this proposed docking mode given the structural similarity of these two agonists (Figure 2B). Definitive structural data, however, are needed to validate this proposed binding mode and any existence of a halogen bond. **19d** was also docked to an active state of 5-HT_2B_R (PDB code 7SRR; orthosteric ligand: LSD)^46^ in the same manner (Figure 2C,D). Given that *des*-bromo analog **19a** exhibits nearly identical activity at 5-HT_2B_ compared to **19d** (EC_50_ = 16 nM vs 14 nM, respectively), it follows that there were no docking results indicative of a potential halogen bond between the bromine of **19d** and the backbone carbonyls of Phe217^5.38^ or Met218^5.39^. Rather, the activity at this receptor is likely driven by a combination of ionic interactions, hydrogen bonding, and π–π interactions.

**Figure 2:**
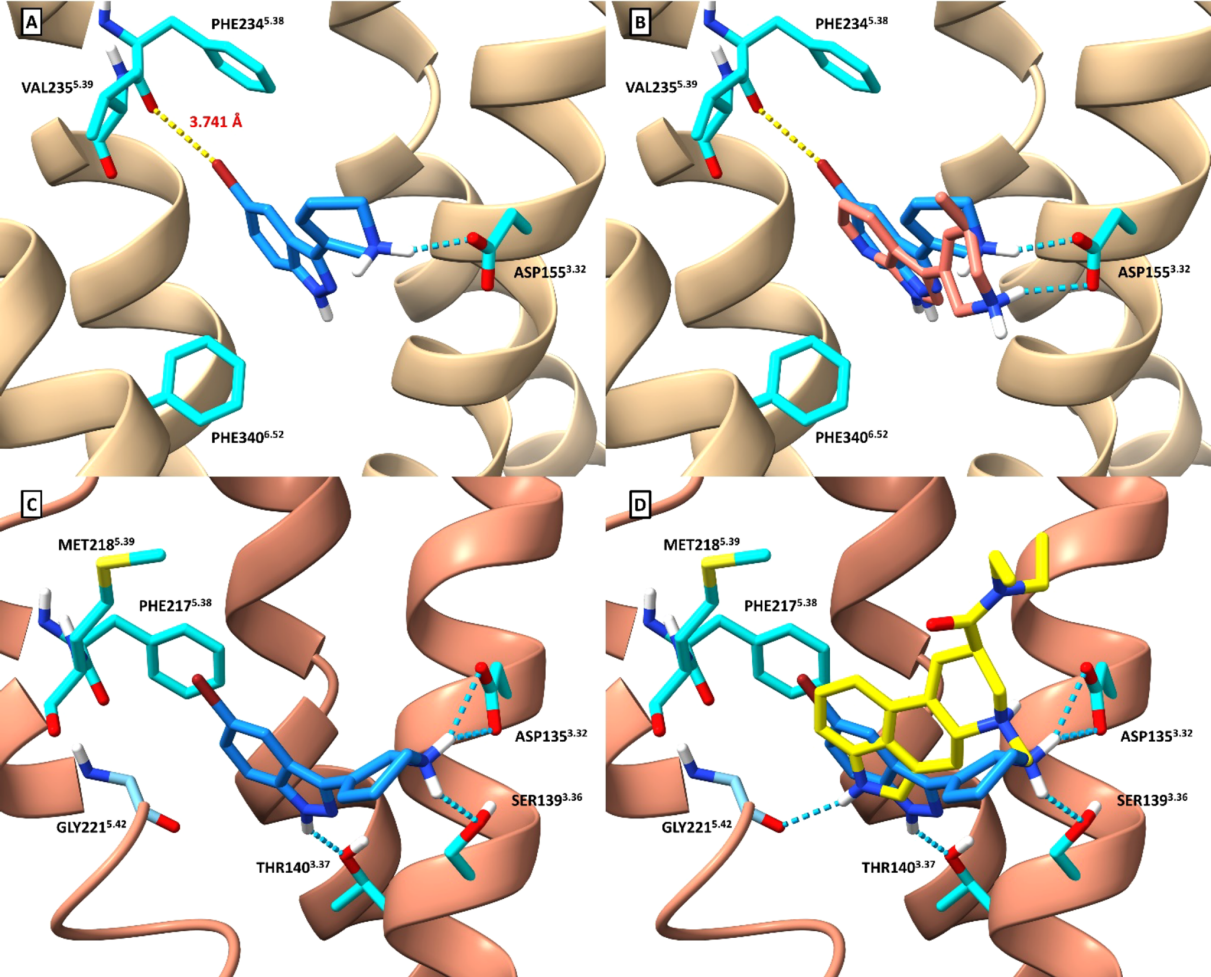
(A) Compound **19d** docked to 5-HT_2A_R, with predicted halogen bonding interaction (yellow dashes) with Phe234^5.38^. (B) Compound **19d** docking pose overlaid with **(*R*)-69** (pink) cryo-EM pose. (C) Compound **19d** docked to 5-HT_2B_R. (D) Compound **19d** 5-HT_2B_R docking pose overlaid with the **LSD** (yellow) cryo-EM pose. Blue dashes depict salt bridges and hydrogen bonds.

In summary, we report herein a novel series of 5-HT_2_ agonists containing an indazole core in place of the traditionally indole-containing tryptamines and show that while many compounds in this series are 5-HT_2A_ agonists, selectivity relative to the other 5-HT_2_ subtypes remains difficult to achieve (and needs to be rigorously profiled, particularly for safety concerns related to 5-HT_2B_ agonism). Although nonselective, VU6067416 (**19d**) is a potent 5-HT_2A_ agonist (possibly due in part to a halogen bonding interaction with Phe234^5.38^ in the 5-HT_2A_ orthosteric pocket), with favorable PK properties for systemic dosing in rats, and is predicted to be brain-penetrant in human. It is our hope that these results will serve to inform the development of next-generation modulators for the 5-HT_2A_ receptor.

## Supporting Information Available

Experimental procedures and characterization for new compounds; experimental details for all assays (PDF)
