# Supplementary material for: Evaluation of the Indazole Analogs of 5-MeO-DMT and Related Tryptamines as Serotonin Receptor 2 Agonists
Source: ACS Med Chem Lett. 2024 Jan 19;15(2):302–9. doi: 10.1021/acsmedchemlett.3c00566 (PMC10860182; doi:10.1021/acsmedchemlett.3c00566)
Supplement: Supplementary file 1 — ml3c00566_si_001.pdf [file ml3c00566_si_001.pdf]

Supporting Information

Evaluation of the Indazole Analogs of 5-MeO-DMT and Related Tryptamines as Serotonin Receptor 2 Agonists

Navoda Jayakodiarachchi,[a,b] Mallory A. Maurer,[a,b] Daniel C. Schultz,[a,b] Cayden J. Dodd,[a,b] Analisa Thompson Gray,[a,b] Hyekyung P. Cho,[a,b] Olivier Boutaud,[a,b] Carrie K. Jones,[a,b] Craig W. Lindsley,[a,b,c,d] Aaron M. Bender,[a,b,*]

*Corresponding author email: aaron.bender@vanderbilt.edu

a. *Warren Center for Neuroscience Drug Discovery, Vanderbilt University, Nashville, Tennessee 37232*
b. *Department of Pharmacology, Vanderbilt University, Nashville, Tennessee 37232*
c. *Department of Chemistry, Vanderbilt University, Nashville, Tennessee 37232*
d. Department of Biochemistry, *Vanderbilt University, Nashville, Tennessee 37232*

**Chemistry**	**S2**
General Experimental Procedures	S2
General Procedure A (amide formation; intermediates **5a, 5b, 13**)	S2
General Procedure B ($LiAlH_4$ or $LiAlD_4$ reduction; final compounds **6a-c, 14, 16**)	S4
Synthesis of Final Compound **11**	S5
General Procedure C (Suzuki-Miyaura coupling; intermediates **18a-18f**)	S7
General Procedure D (Boc-deprotection; final compounds **19a-19f**)	S9
Synthesis of Final Compound **20**	S10
Synthesis of Final Compound **21**	S11
Copies of NMR Spectra and LCMS Trace for Key Compound **19d**	S12
**Molecular Pharmacology**	**S14**
**DMPK**	**S16**
**Docking Experiments**	**S20**

General Synthetic Methods

All reactions were carried out employing standard chemical techniques. Solvents used for extraction, washing, and chromatography were HPLC grade. All reagents were purchased from commercial sources and were used without further purification.

All NMR spectra were recorded on a 400 MHz Bruker AV-400 instrument. ^{1}H chemical shifts are reported as δ values in ppm relative to the residual solvent peak ($CDCl_3$ = 7.26, MeOD-d_4 = 3.31). Data are reported as follows: chemical shift, multiplicity (br = broad, s = singlet, d = doublet, t = triplet, q = quartet, p = pentet, dd = doublet of doublets, ddd = doublet of doublet of doublets, td = triplet of doublets, m = multiplet), coupling constant, and integration. ^{13}C chemical shifts are reported as δ values in ppm relative to the residual solvent peak ($CDCl_3$ = 77.16, MeOD-d_4 = 49.0).

Low-resolution mass spectra were obtained on a Waters QDa (Performance) SQ MS with ESI source. MS parameters were as follows: cone voltage: 15 V, capillary voltage: 0.8 kV, probe temperature: 600° C. Samples were introduced via an Acquity I-Class PLUS UPLC comprised of a BSM, FL-SM, CH-A, and PDA. UV absorption was generally observed at 215 nm and 254 nm; 4 nm bandwidth.

High resolution mass spectra were obtained on an Agilent 6540 UHD Q-TOF with ESI source. MS parameters were as follows: fragmentor: 150, capillary voltage: 3500 V, nebulizer pressure: 60 psig, drying gas flow: 13 L/min, drying gas temperature: 275° C. Samples were introduced via an Agilent 1290 UHPLC comprised of a G4220A binary pump, G4226A ALS, G1316C TCC, and G4212A DAD with ULD flow cell. UV absorption was observed at 215 nm and 254 nm with a 4 nm bandwidth. Column: Agilent Zorbax Extend C18, 1.8 µm, 2.1 x 50 mm. Gradient conditions: 5% to 95% CH_3CN in H_2O (0.1% formic Acid) over 1 min, hold at 95% CH_3CN for 0.1 min, 0.5 mL/min, 40° C.

Automated flash column chromatography was performed on a Biotage Isolera 1 or a Teledyne ISCO CombiFlash system. RP-HPLC was performed on a Gilson preparative reversed-phase HPLC system comprised of a 333 aqueous pump with solvent-selection valve, 334 organic pump, GX-271 or GX-281 liquid hander, two column switching valves, and a 155 UV detector. Absorbance was typically monitored at 215 or 220 nm. Column: Phenomenex Axia-packed Gemini C18, 5 µm. Mobile phase: CH_3CN in H_2O (0.1% TFA) or CH_3CN in H_2O (0.05% *v/v* NH_4OH) under the specified gradient, then hold 95% CH_3CN in 5% aqueous phase, 50 mL/min, 23° C. All compounds were found to be >95% pure by LCMS analysis.

Safety statement*: no unexpected or unusually high safety hazards were encountered. Dess-Martin periodinane (DMP) should be handled with care, particularly in solid form, and should be used as a solution when possible.*

General Procedure A (amide formation; intermediates 5a, 5b, 13)

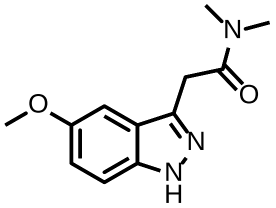

(5a) 2-(5-Methoxy-1*H*-indazol-3-yl)-*N,N*-dimethylacetamide. To a solution of methyl 2-(5-methoxy-1*H*-indazol-3-yl)acetate (95 mg, 0.43 mmol, 1 eq) in THF (1.5 mL) and H_2O (1.5 mL)

was added LiOH (31 mg, 1.29 mmol, 3 eq). The resulting reaction mixture was stirred at r.t. for 1 h, after which time solvents were concentrated, and the resulting lithium carboxylate was dried under vacuum and used without further purification (91.5 mg, 100%). To a solution of the lithium carboxylate (61 mg, 0.29 mmol, 1 eq) and dimethylamine hydrochloride (70 mg, 0.86 mmol, 3 eq) in DMF (2 mL) was added DIPEA (0.25 mL, 1.44 mmol, 5 eq) and HATU (164 mg, 0.43 mmol, 1.5 eq). The resulting reaction mixture was stirred at r.t. for 1 h, after which time the reaction mixture was purified directly by RP-HPLC (5-45% MeCN in 0.05% NH_4OH solution over 5 min). Fractions were concentrated to give the title compound as a colorless oil (44 mg, 65%). ^1H NMR (400 MHz, $CDCl_3$) δ 7.34 (d, *J* = 9.0 Hz, 1H), 7.20 (d, *J* = 2.3 Hz, 1H), 7.06 (dd, *J* = 9.0, 2.4 Hz, 1H), 4.07 (s, 2H), 3.86 (s, 3H), 3.12 (s, 3H), 2.97 (s, 3H); $C_{12}H_{16}N_3O_2$ $[M+H]^+$ calc. mass 234.1, found 234.2.

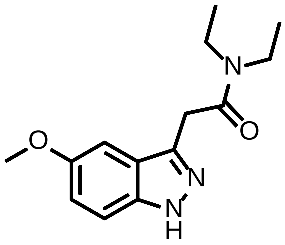

(5b) *N,N*-Diethyl-2-(5-methoxy-1*H*-indazol-3-yl)acetamide. Prepared in a similar manner from lithium 2-(5-methoxy-1*H*-indazol-3-yl)acetate (31 mg, 0.14 mmol) and diethylamine (0.045 mL, 0.43 mmol) to give the title compound as a colorless oil (22 mg, 58%). $C_{14}H_{20}N_3O_2$ $[M+H]^+$ calc. mass 262.2, found 262.2.

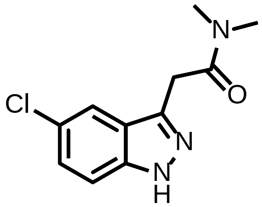

(13) 2-(5-Chloro-1*H*-indazol-3-yl)-*N,N*-dimethylacetamide. Prepared in a similar manner from lithium 2-(5-chloro-1*H*-indazol-3-yl)acetate (103 mg, 0.47 mmol) and dimethylamine hydrochloride (116 mg, 1.42 mmol) to give the title compound as a colorless oil (29 mg, 26%). $C_{11}H_{13}ClN_3O$ $[M+H]^+$ calc. mass 238.1, found 238.0.

General Procedure B ($LiAlH_4$ or $LiAlD_4$ reduction; final compounds 6a-c, 14, 16)

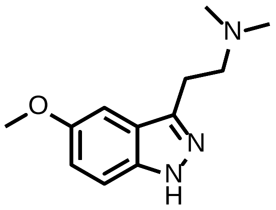

(6a) 2-(5-Methoxy-1*H*-indazol-3-yl)-*N,N*-dimethylethan-1-amine. To a solution of lithium aluminum hydride (0.14 mL, 0.28 mmol, 3 eq, 2M solution in THF) in THF (0.5 mL) was added a solution of **5a** (22 mg, 0.094 mmol, 1 eq) in THF (0.5 mL) at r.t. The resulting reaction mixture was stirred at r.t. under an inert atmosphere for 30 min, after which time the reaction mixture was diluted with DCM and quenched with the slow addition of sat. $NaHCO_3$ solution. The aqueous layer was extracted with DCM, and combined organic extracts were filtered through a phase separator and concentrated. Crude residue was purified by RP-HPLC (2-32% MeCN in 0.1% aqueous TFA solution over 5 min). Fractions containing product were basified with sat. $NaHCO_3$ solution, and extracted with DCM. Combined organic extracts were filtered through a phase separator and concentrated to give the title compound as a white solid (2.3 mg, 11%). ^{1}H NMR (400 MHz, $CDCl_3$) δ 7.32 (dd, J = 8.5, 1.1 Hz, 1H), 7.06 – 7.03 (m, 2H), 3.86 (s, 3H), 3.16 – 3.12 (m, 2H), 2.80 – 2.76 (m, 2H), 2.36 (s, 6H); $C_{12}H_{18}N_3O$ $[M+H]^+$ calc. mass 220.1, found 220.2.

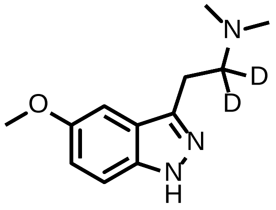

(6b) 2-(5-Methoxy-1*H*-indazol-3-yl)-*N,N*-dimethylethan-1-amine-1,1-d_2. Prepared in a similar manner from **5a** (22 mg, 0.094 mmol) using lithium aluminum deuteride (11 mg, 0.28 mmol) to give the title compound as a white solid (2.7 mg, 13%). $C_{12}H_{16}D_2N_3O$ $[M+H]^+$ calc. mass 222.2, found 222.1.

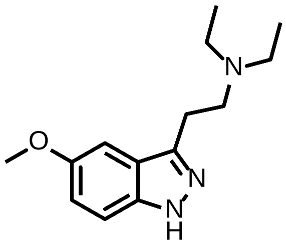

(6c) *N,N*-Diethyl-2-(5-methoxy-1*H*-indazol-3-yl)ethan-1-amine. Prepared in a similar manner from **5b** (22 mg, 0.084 mmol) to give the title compound as a colorless oil (4.0 mg, 19%). $C_{14}H_{22}N_3O$ $[M+H]^+$ calc. mass 248.2, found 248.1.

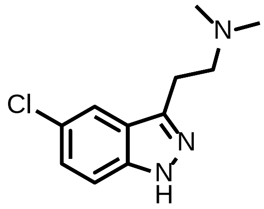

(14) 2-(5-Chloro-1*H*-indazol-3-yl)-*N,N*-dimethylethan-1-amine. Prepared in a similar manner from **13** (29 mg, 0.12 mmol) to give the title compound as a colorless oil (3.9 mg, 14%). $C_{11}H_{15}ClN_3$ $[M+H]^+$ calc. mass 224.1, found 224.1.

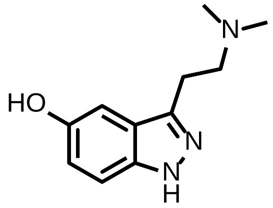

(16) 3-(2-(Dimethylamino)ethyl)-1*H*-indazol-5-ol. Prepared in a similar manner with the following modification: 2-(5-hydroxy-1H-indazol-3-yl)acetic acid (50 mg, 0.26 mmol, 1 eq) and dimethylamine hydrochloride (64 mg, 0.78 mmol, 3 eq) were combined in THF (1.5 mL) and DMF (0.5 mL), and DIPEA (0.23 mL, 1.30 mmol, 5 eq) was added, followed by HATU (119 mg, 0.31 mmol, 1.2 eq). The resulting reaction mixture was stirred at r.t. for 1 h, after which time the reaction mixture was diluted with sat. $NaHCO_3$ solution, and extracted with 3:1 chloroform/IPA solution (*v/v*). The combined organic extracts were filtered through a phase separator and concentrated, and the crude dimethyl amide was dried under vacuum and used directly without further purification (57 mg, 100%). The crude amide precursor (57 mg, 0.26 mmol) was then subjected to the LAH reduction conditions described in General Procedure B to give the title compound as a tan oil (6.1 mg, 11%). $C_{11}H_{16}N_3O$ $[M+H]^+$ calc. mass 206.1, found 206.2.

Synthesis of Final Compound 11

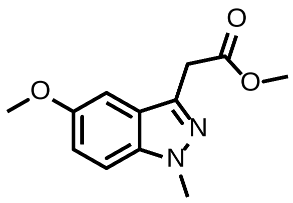

(8) Methyl 2-(5-methoxy-1-methyl-1*H*-indazol-3-yl)acetate. To a suspension of 2-(5-hydroxy-1*H*-indazol-3-yl)acetic acid (200 mg, 1.04 mmol, 1 eq) and cesium carbonate (1.7 g, 5.20 mmol, 5 eq) in DMF (5 mL) was added iodomethane (0.32 mL, 5.20 mmol, 5 eq) dropwise under an inert atmosphere. The resulting reaction mixture was stirred at r.t. overnight, after which time the reaction mixture was diluted with EtOAc and H_2O, and the organic layer was washed with H_2O (2x) and brine (1x), and dried over $MgSO_4$. Solvents were filtered and concentrated, and crude residue was purified by column chromatography (3-100% EtOAc in hexanes) to give the title compound as a colorless oil (66.7 mg, 27%). ^1H NMR (400 MHz, $CDCl_3$) δ 7.27 – 7.24 (m, 1H), 7.09 – 7.05 (m, 1H), 7.01 (dd, *J* = 2.4, 0.6 Hz, 1H), 4.02 – 3.98 (m, 5H), 3.86 (s, 3H), 3.72 (s, 3H); $C_{12}H_{15}N_2O_3$ $[M+H]^+$ calc. mass 235.1, found 235.2.

*N*1-indazole methylation assigned by comparison of chemical shift to literature NMR spectra of analogous methylated 2-(indazol-3-yl)acetates.[1-3]

1. Yao, J.; Feng, R.; Lin, C.; Liu, Z.; Zhang, Y. Synthesis of 2,3-Dihydro-1*H*-Indazoles by Rh(III)-Catalyzed C-H Cleavage of Arylhydrazines. *Org. Biomol. Chem*. **2014**, *12*, 5469-5476.
2. Han, S.; Shin, Y.; Sharma, S.; Mishra, N. K.; Park. J.; Kim, M.; Kim, M.; Jang, J.; Kim, I. S. Rh(III)-Catalyzed Oxidative Coupling of 1,2-Disubstituted Arylhydrazines and Olefins: A New Strategy for 2,3-Dihydro-1*H*-Indazoles. *Org. Lett*. **2014**, *16*, 2494-2497.
3. Zhao, D.; Vásquez-Céspedes, S.; Glorius, F. Rhodium(III)-Catalyzed Cyclative Capture Approach to Diverse 1-Aminoindoline Derivatives at Room Temperature. *Angew. Chem. Int. Ed*. **2015**, *54*, 1657-1661.

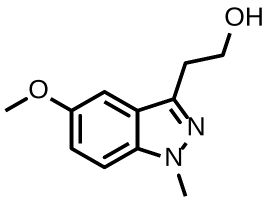

(9) 2-(5-Methoxy-1-methyl-1*H*-indazol-3-yl)ethan-1-ol. To a solution of **8** (67 mg, 0.28 mmol, 1 eq) in DCM (3 mL) was added DIBAL (0.57 mL, 0.57 mmol, 2 eq, 1.0M solution in toluene) at -78 °C under an inert atmosphere. The resulting reaction mixture was slowly warmed to r.t. and stirred overnight, after which time the reaction mixture was cooled back to 0 °C, and quenched with the sequential addition of H_2O (23 µL), 4M NaOH solution (23 µL), and H_2O (57 µL). The reaction mixture was warmed to r.t. and stirred for 15 min, after which time $MgSO_4$ was added, followed by an additional 15 min of stirring. Solids were removed by filtration with DCM, and the filtrate was concentrated to give a slightly yellow solid, which was dried under vacuum and used without further purification (39 mg, 67%; ~1.8 to 1 mixture of alcohol and aldehyde). $C_{11}H_{15}N_2O_2$ $[M+H]^+$ calc. mass 207.1, found 207.1.

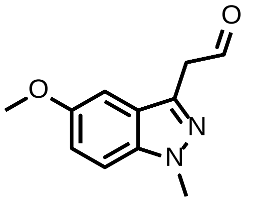

(10) 2-(5-Methoxy-1-methyl-1*H*-indazol-3-yl)acetaldehyde. To a suspension of the 2-(5-methoxy-1-methyl-1*H*-indazol-3-yl)ethan-1-ol and 2-(5-methoxy-1-methyl-1*H*-indazol-3-yl)acetaldehyde mixture **9** (39 mg, 0.19 mmol, 1 eq) in DCM (2 mL) was added Dess-Martin periodinane (96 mg, 0.23 mmol, 1.2 eq). The resulting reaction mixture was stirred at r.t. for 1 h, after which time the reaction mixture was quenched with the addition of sat. sodium thiosulfate solution and sat. $NaHCO_3$ solution, and stirred for 30 min. The aqueous layer was extracted with DCM, and combined organic extracts were filtered through a phase separator and concentrated to give the title compound as a yellow oil, which was dried under vacuum and used without additional purification (35.1 mg, 91%). $C_{11}H_{13}N_2O_2$ $[M+H]^+$ calc. mass 205.1, found 205.1.

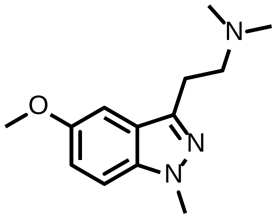

(11) 2-(5-Methoxy-1-methyl-1*H*-indazol-3-yl)-*N,N*-dimethylethan-1-amine. To a solution of **10** (18 mg, 0.086 mmol, 1 eq) and dimethylamine hydrochloride (35 mg, 0.43 mmol, 5 eq) in DCM (1 mL) was added sodium triacetoxyborohydride (91 mg, 0.43 mmol, 5 eq). The resulting reaction mixture was stirred at r.t. for 1 h, after which time the reaction mixture was quenched with the addition of sat. $NaHCO_3$ solution, and extracted with DCM. Combined organic extracts were filtered through a phase separator and concentrated. Crude residue was purified by RP-HPLC (2-32% MeCN in 0.1% aqueous TFA solution over 5 min). Fractions containing product were basified with sat. $NaHCO_3$ solution, and extracted with DCM. Combined organic extracts were filtered through a phase separator and concentrated to give the title compound as a colorless oil (6.6 mg, 33%). ^{1}H NMR (400 MHz, $CDCl_3$) δ 7.23 (dd, J = 9.0, 0.7 Hz, 1H), 7.05 (dd, J = 9.0, 2.3 Hz, 1H), 7.02 – 7.00 (m, 1H), 3.98 (s, 3H), 3.86 (s, 3H), 3.13 – 3.08 (m, 2H), 2.76 – 2.71 (m, 2H), 2.35 (s, 6H); $C_{13}H_{20}N_3O$ $[M+H]^+$ calc. mass 234.2, found 234.3.

General Procedure C (Suzuki-Miyaura coupling; intermediates 18a-18f)

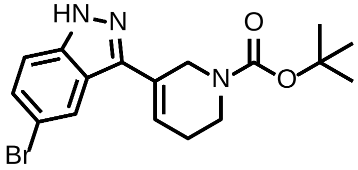

(18d) *Tert*-butyl 5-(5-bromo-1*H*-indazol-3-yl)-3,6-dihydropyridine-1(2*H*)-carboxylate. 5-Bromo-3-iodo-1*H*-indazole (60 mg, 0.19 mmol, 1 eq), *tert*-butyl 5-(4,4,5,5-tetramethyl-1,3,2-dioxaborolan-2-yl)-3,6-dihydropyridine-1(2*H*)-carboxylate (86 mg, 0.28 mmol, 1.5 eq), K_2CO_3 (78 mg, 0.56 mmol, 3 eq), and dichloro[1,1'-bis(diphenylphosphino)ferrocene]palladium(II) dichloromethane adduct (15 mg, 0.019 mmol, 0.1 eq) were suspended in 1,4-dioxane/H_2O (10:1 ratio, 1.65 mL total) at r.t. The resulting reaction mixture was stirred at 110 °C for 2 h under an inert atmosphere, after which time the reaction mixture was cooled to r.t and was diluted with DCM and water. The aqueous layer was extracted with DCM, and combined organic extracts were filtered through a phase separator and concentrated. The crude residue was purified by column chromatography (3-100% EtOAc in hexanes) and fractions were concentrated to give the title compound as a white solid. (41 mg, 58%). ^{1}H NMR (400 MHz, MeOD-d_4) δ 8.07 (dd, J = 1.7, 0.8 Hz, 1H), 7.48 – 7.42 (m, 2H), 6.63 – 6.60 (m, 1H), 4.45 (q, J = 2.4 Hz, 2H), 3.62 (t, J = 5.8 Hz, 2H), 2.44 – 2.39 (m, 2H), 1.51 (s, 9H). $C_{17}H_{21}BrN_3O_2$ $[M+H]^+$ calc. mass 378.1, found 322.1, 324.1 (- t-butyl).

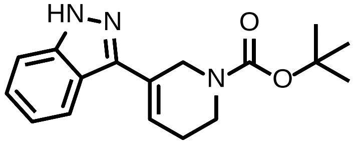

(18a) *Tert*-butyl 5-(1*H*-indazol-3-yl)-3,6-dihydropyridine-1(2*H*)-carboxylate. Prepared in a similar manner from 3-bromo-1*H*-indazole (0.5 g, 2.54 mmol, 1 eq) to give the title compound as a white solid. (0.55 g, 66%). $C_{17}H_{22}N_3O_2$ $[M+H]^+$ calc. mass 300.2, found 244.2 (- t-butyl).

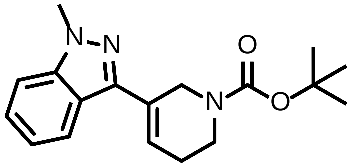

(18b) *Tert*-butyl 5-(1-methyl-1*H*-indazol-3-yl)-3,6-dihydropyridine-1(2*H*)-carboxylate. Prepared in a similar manner from 3-bromo-1-methyl-1*H*-indazole (40 mg, 0.19 mmol, 1 eq) to give the title compound as a white solid. (28 mg, 47%). $C_{18}H_{24}N_3O_2$ $[M+H]^+$ calc. mass 314.2, found 258.3 (- t-butyl).

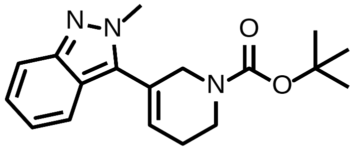

(18c) *Tert*-butyl 5-(2-methyl-2*H*-indazol-3-yl)-3,6-dihydropyridine-1(2*H*)-carboxylate. Prepared in a similar manner from 3-bromo-2-methyl-2*H*-indazole (40 mg, 0.19 mmol, 1 eq) to give the title compound as a colorless oil (39 mg, 65%). $C_{18}H_{24}N_3O_2$ $[M+H]^+$ calc. mass 314.2, found 314.1.

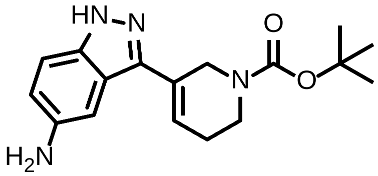

(18e) *Tert*-butyl 5-(5-amino-1*H*-indazol-3-yl)-3,6-dihydropyridine-1(2*H*)-carboxylate. Prepared in a similar manner from 3-bromo-1*H*-indazol-5-amine (40 mg, 0.19 mmol, 1 eq) to give the title compound as a white solid. (24 mg, 41%). $C_{17}H_{23}N_4O_2$ $[M+H]^+$ calc. mass 315.2, found 259.2 (- t-butyl).

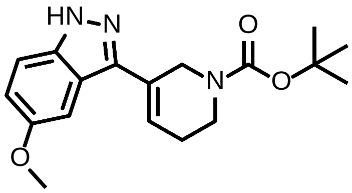

(18f) *Tert*-butyl 5-(5-methoxy-1*H*-indazol-3-yl)-3,6-dihydropyridine-1(2*H*)-carboxylate. Prepared in a similar manner from 3-bromo-5-methoxy-1*H*-indazole (50 mg, 0.22 mmol, 1 eq), to give the title compound as a white solid (20 mg, 28%). $C_{18}H_{24}N_3O_3$ $[M+H]^+$ calc. mass 330.2, found 274.2 (- t-butyl).

General Procedure D (Boc-deprotection; final compounds 19a-19f)

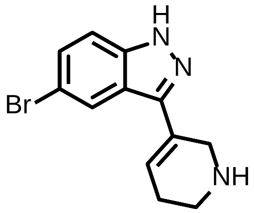

(19d) 5-Bromo-3-(1,2,5,6-tetrahydropyridin-3-yl)-1*H*-indazole (VU6067416). To a solution of **18d** (37 mg, 0.097 mmol, 1 eq) in DCM (1.5 mL) was added hydrochloric acid (0.24 mL, 0.96 mmol, 10 eq, 4M in 1,4-dioxane) at r.t. The resulting reaction mixture was stirred at r.t for 2 h, after which time the solvents were concentrated and crude residue was diluted with DCM and quenched with the slow addition of sat. $NaHCO_3$ solution. The aqueous layer was extracted with 3:1 chloroform/IPA solution (*v/v*), and combined organic extracts were filtered through a phase separator and concentrated to give the title compound as a white solid (24 mg, 90%). ^{1}H NMR (400 MHz, MeOD-d_4) δ 8.08 – 8.06 (m, 1H), 7.47 – 7.42 (m, 2H), 6.60 – 6.57 (m, 1H), 3.83 (q, *J* = 2.4 Hz, 2H), 3.02 (t, *J* = 5.8 Hz, 2H), 2.42 – 2.37 (m, 2H); ^{13}C NMR (101 MHz, MeOD-d_4) δ 144.6, 141.6, 131.8, 130.6, 126.6, 124.7, 122.9, 114.9, 113.0, 46.3, 43.2, 26.3. $C_{12}H_{13}BrN_3$ $[M+H]^+$ calc. mass 278.0287, found 278.0289.

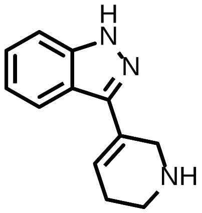

(19a) 3-(1,2,5,6-Tetrahydropyridin-3-yl)-1*H*-indazole. Prepared in a similar manner from **18a** (200 mg, 0.67 mmol, 1 eq) to give the title compound as a white solid (127 mg, 95%). $C_{12}H_{14}N_3$ $[M+H]^+$ calc. mass 200.1, found 200.2.

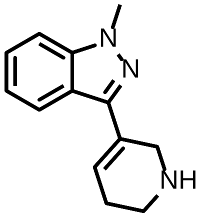

(19b) 1-Methyl-3-(1,2,5,6-tetrahydropyridin-3-yl)-1*H*-indazole. Prepared in a similar manner from **18b** (20 mg, 0.064 mmol, 1 eq) to give the title compound as a white solid (10 mg, 73%). $C_{13}H_{16}N_3$ $[M+H]^+$ calc. mass 214.1, found 214.1.

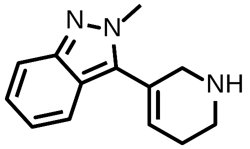

(19c) 2-Methyl-3-(1,2,5,6-tetrahydropyridin-3-yl)-2*H*-indazole. Prepared in a similar manner from **18c** (30 mg, 0.096 mmol, 1 eq) to give the title compound as a white solid (8.6 mg, 42%). $C_{13}H_{16}N_3$ $[M+H]^+$ calc. mass 214.1, found 214.1.

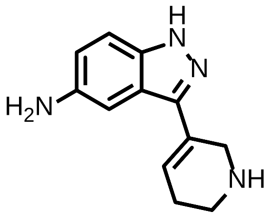

(19e) 3-(1,2,5,6-Tetrahydropyridin-3-yl)-1*H*-indazol-5-amine. Prepared in a similar manner from **18e** (20 mg, 0.064 mmol, 1 eq) to give the title compound as a white solid (4.8 mg, 35%). $C_{12}H_{15}N_4$ $[M+H]^+$ calc. mass 215.1, found 215.1.

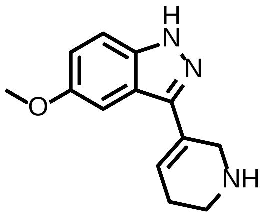

(19f) 5-Methoxy-3-(1,2,5,6-tetrahydropyridin-3-yl)-1*H*-indazole. Prepared in a similar manner from **18f** (17 mg, 0.052 mmol, 1 eq) to give the title compound as a white solid (1.4 mg, 12%). $C_{13}H_{16}N_3O$ $[M+H]^+$ calc. mass 230.1, found 230.3.

Synthesis of Final Compound 20

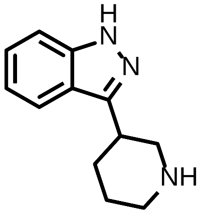

(20) 3-(Piperidin-3-yl)-1*H*-indazole. To a solution of **18a** (200 mg, 0.67 mmol, 1 eq) in MeOH (2 mL) was added ammonium formate (97 mg, 1.5 mmol, 2.3 eq), followed by 10% palladium on activated carbon (71 mg, 1.5 mmol, 0.1 eq). The resulting reaction mixture was stirred at reflux under an N_2 atmosphere for 4 h, after which time the reaction mixture was cooled to r.t and filtered over a pad of Celite with MeOH. The filtrate was concentrated to give the intermediate *tert*-butyl 3-(1*H*-indazol-3-yl)piperidine-1-carboxylate as a white solid (169 mg, 83%). $C_{17}H_{24}N_3O_2$ $[M+H]^+$ calc. mass 302.2, found 302.2.

Tert-butyl 3-(1*H*-indazol-3-yl)piperidine-1-carboxylate (140 mg, 0.46 mmol, 1 eq) was taken up in DCM (2.5 mL), and hydrochloric acid (1.2 mL, 4.7 mmol, 10 eq, 4M in 1,4-dioxane) was added at r.t. The resulting reaction mixture was stirred at r.t for 2 h, after which time the mixture was concentrated and the crude residue was diluted with DCM and quenched with the slow addition of sat. $NaHCO_3$ solution. The aqueous layer was extracted with 3:1 chloroform/IPA solution (*v/v*), and combined organic extracts were filtered through a phase separator and concentrated to give the title compound as a white solid (91 mg, 97%). ^1H NMR (400 MHz, $CDCl_3$) δ 7.77 – 7.68 (m, 1H), 7.44 – 7.39 (m, 1H), 7.33 – 7.27 (m, 1H), 7.10 – 7.04 (m, 1H), 3.41 – 3.22 (m, 2H), 3.17 – 2.95 (m, 2H), 2.79 – 2.68 (m, 1H), 2.25 – 2.14 (m, 1H), 2.02 – 1.88 (m, 1H), 1.85 – 1.54 (m, 2H); $C_{12}H_{16}N_3$ $[M+H]^+$ calc. mass 202.1, found 202.3.

Synthesis of Final Compound 21

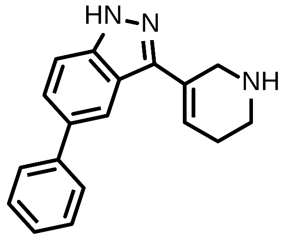

(21) 5-Phenyl-3-(1,2,5,6-tetrahydropyridin-3-yl)-1*H*-indazole. Compound **18d** (30 mg, 0.079 mmol, 1 eq), phenylboronic acid (15 mg, 0.12 mmol, 1.5 eq), K_2CO_3 (33 mg, 0.24 mmol, 3 eq), and dichloro[1,1'-bis(diphenylphosphino)ferrocene]palladium(II) dichloromethane adduct (6.5 mg, 0.008 mmol, 0.1 eq) were suspended in 1,4-dioxane/H_2O (10:1 ratio, 1.1 mL total) at r.t. The resulting reaction mixture was stirred at 110 °C for 2 h under an inert atmosphere, after which time the reaction mixture was cooled to r.t and was diluted with DCM and H_2O. The aqueous layer was extracted with DCM, and combined organic extracts were filtered through a phase separator and concentrated. Crude residue was purified by RP-HPLC (30-95% MeCN in 0.05% aqueous NH_4OH solution over 7 min). Fractions containing product were concentrated to give the intermediate *tert*-butyl 5-(5-phenyl-1*H*-indazol-3-yl)-3,6-dihydropyridine-1(2*H*)-carboxylate as a white solid. (12 mg, 40%). $C_{23}H_{26}N_3O_2$ $[M+H]^+$ calc. mass 376.2, found 320.1 (- t-butyl).

To a solution of *tert*-butyl 5-(5-phenyl-1*H*-indazol-3-yl)-3,6-dihydropyridine-1(2*H*)-carboxylate (10 mg, 0.027 mmol, 1 eq) in DCM (0.5 mL) was added hydrochloric acid (67 µL, 0.27 mmol, 10 eq, 4M in 1,4-dioxane) at r.t. The resulting reaction mixture was stirred at r.t for 2 h, after which time the solvents were concentrated and crude residue was diluted with DCM and quenched with the slow addition of sat. $NaHCO_3$ solution. The aqueous layer was extracted with 3:1 chloroform/IPA solution (*v/v*), and combined organic extracts were filtered through a phase separator and concentrated to give the title compound as a white solid (2.6 mg, 35%). ^1H NMR (400 MHz, MeOD-d_4) δ 8.07 (s, 1H), 7.68 – 7.60 (m, 3H), 7.56 (d, J = 8.7 Hz, 1H), 7.44 (t, J = 7.6 Hz, 2H), 7.32 (t, J = 7.4 Hz, 1H), 6.73 – 6.67 (m, 1H), 3.90 – 3.83 (m, 2H), 3.03 (t, J = 5.8 Hz, 2H), 2.45 – 2.36 (m, 2H). $C_{18}H_{18}N_3$ $[M+H]^+$ calc. mass 276.1, found 276.4.

Figure S1: Copies of NMR Spectra and LCMS Trace for Key Compound 19d

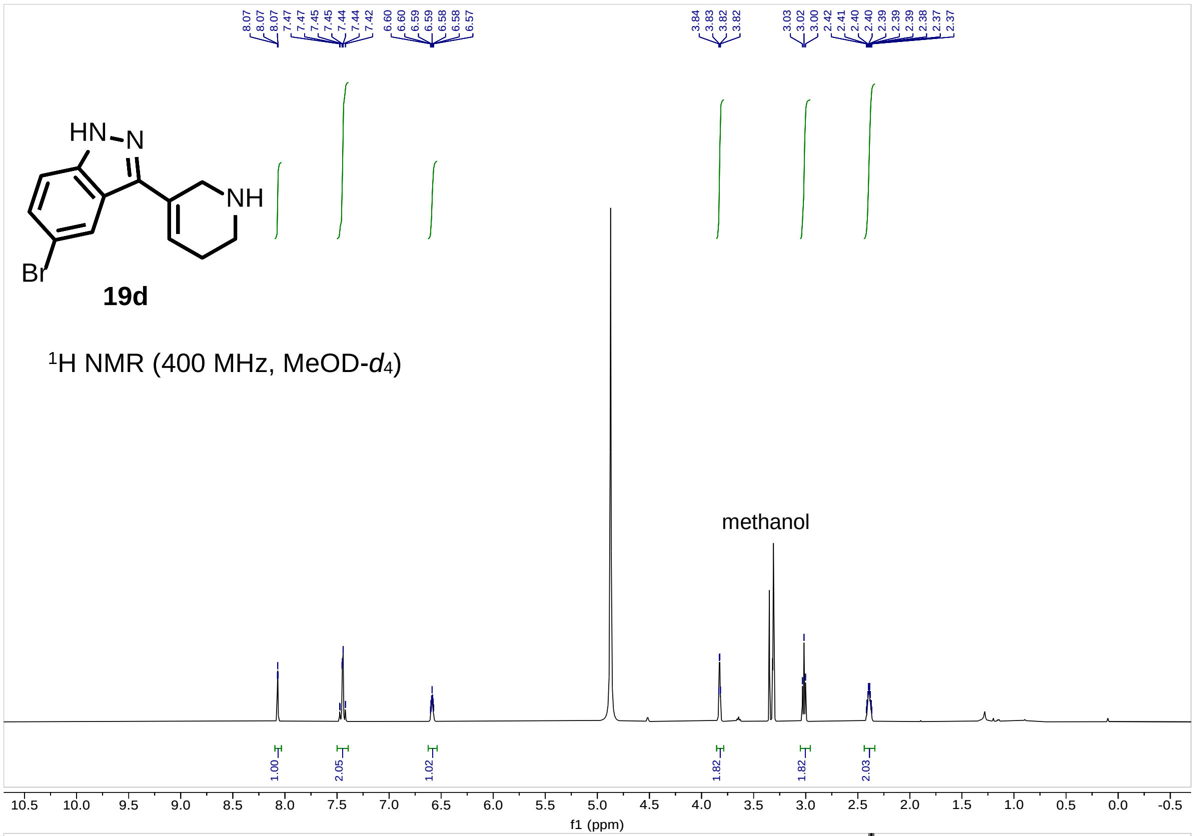

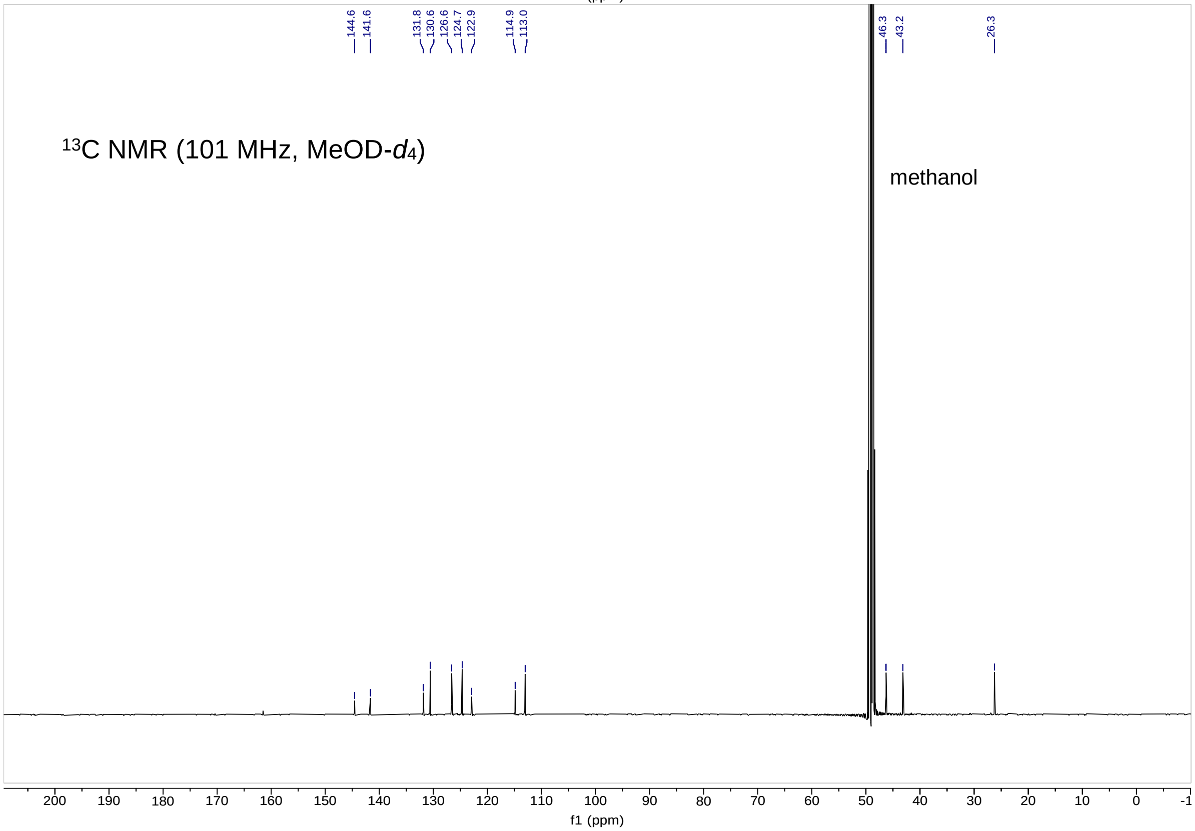

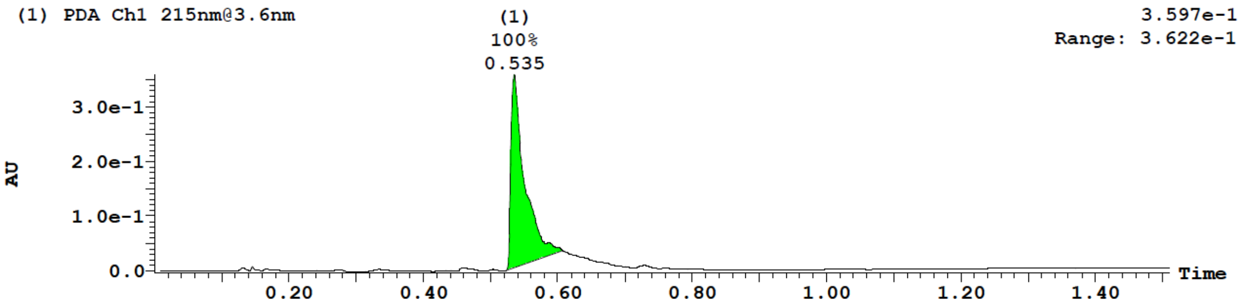

Peak Number	Time	Area %Total	BPM	Mass Found
1	0.535	100.00	280	279.0000

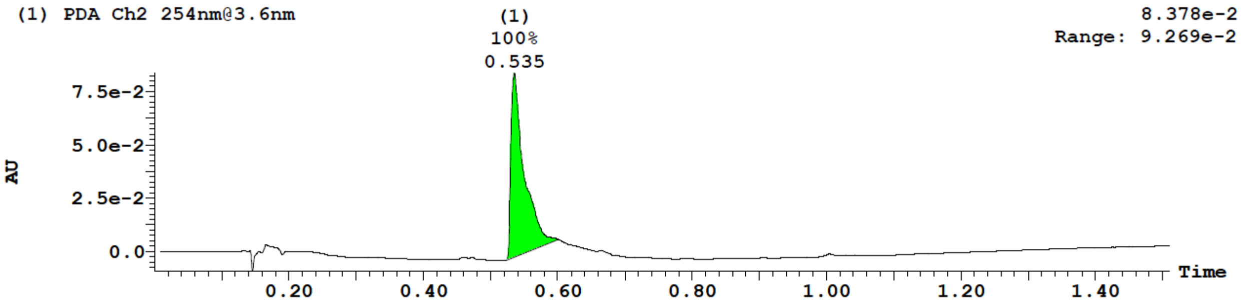

Peak Number	Time	Area %Total	BPM	Mass Found
1	0.535	100.00	280	279.0000

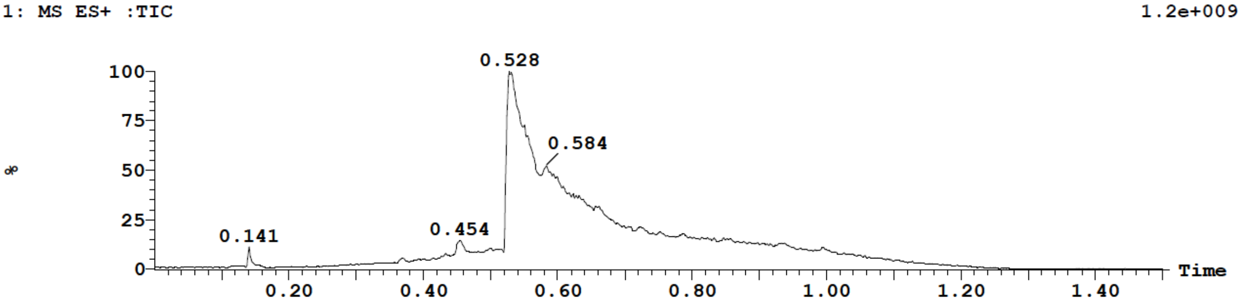

Peak ID	Time
1	0.535

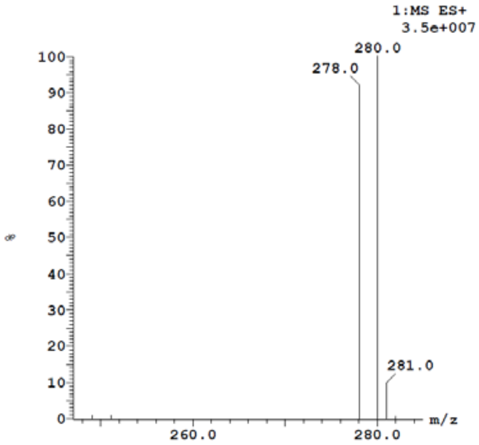

Molecular Pharmacology

5-HT$_{2A}$ Calcium Mobilization Assay. To determine the functional activity of 5-HT$_{2A}$ compounds in a cellular assay, receptor-induced mobilization of intracellular calcium was measured in Chinese Hamster Ovary (CHO) cells stably expressing human 5-HT$_{2A}$. The stable 5-HT$_{2A}$-CHO cells were cultured in F12 medium containing 1% fetal bovine serum, 20 mM HEPES, 100 units/mL antibiotics/antimycotic, and 0.5 mg/ml G418. All reagents used were from Life Technologies (Carlsbad, CA) unless otherwise noted.

The day before the assay, stable 5-HT$_{2A}$-CHO cells (15,000 cells/20 μL/well) were plated in black-walled, clear-bottomed, 384 well plates (Greiner Bio-One, Monroe, NC) in F12 medium containing 10% dialyzed fetal bovine serum, 20 mM HEPES, and 100 units/mL antibiotics/antimycotic and then incubated overnight at 37 °C in the presence of 5% CO_2. The next day, calcium assay buffer (Hank's balanced salt solution (HBSS), 20 mM HEPES, 2.5 mM Probenecid, 4.16 mM sodium bicarbonate (Sigma-Aldrich, St. Louis, MO)) was prepared to dilute compounds, agonists, and Fluo-4-acetomethoxyester (Fluo-4-AM, Ion Biosciences) fluorescent calcium indicator dye. Compounds (10 mM stock in DMSO) were serially diluted 1:3 into 10 point concentration response curves in DMSO using the Bravo Liquid Handler (Agilent, Santa Clara, CA). The diluted compounds were transferred to 384 well daughter plates using an Echo acoustic liquid handler (Beckman Coulter, Indianapolis, Indiana), and diluted in assay buffer to a 2X final concentration.

As follows, screening was conducted using our previously reported "triple-add" protocol.[1] Although compounds were monitored for agonist (no 5-HT addition), PAM and antagonist activity (potentiation of 5-HT addition) in a single run, no potentiation of an EC_{40} 5-HT concentration was noted for any compounds in the present manuscript (no observed positive allosteric modulator (PAM) activity):

The agonist plates were prepared using 5-HT (Tocris, Minneapolis, MN) concentrations to elicit the EC_{40} and EC_{max} responses by diluting in assay buffer to a 5X final concentration. The 2X dye solution (2.3 μM) was prepared by mixing a 2.3 mM Fluo-4-AM stock in DMSO with 10% (*w/v*) pluronic acid F-127 in a 1:1 ratio in assay buffer. Using a microplate washer (BioTek, Winooski, VT), cells were washed with assay buffer 4 times to remove medium. After the final wash, 20 μL of assay buffer remained in the cell plates. Immediately, 20 μL of the 2X dye solution (final 1.15 μM) was added to each well of the cell plate using a Multidrop Combi dispenser (Thermo Fisher, Waltham, MA). After cells were incubated with the dye solutions for 50 min at 37 °C in the presence of 5% CO_2, the dye solutions were removed and replaced with assay buffer using a microplate washer, leaving 20 μL of assay buffer in the cell plate. The compound, agonist, and cell plates were placed inside the Functional Drug Screening System uCell (FDSS uCell, Hamamatsu, Japan) to measure calcium flux. The triple add protocol was used to measure Ca kinetics: compound, 5-HT for EC_{40}, and 5-HT for EC_{max} adds in order. Briefly, after establishment of a fluorescence baseline for 2 seconds (excitation, 480 nm; emission, 530 nm), first add occurred by adding 20 μL of test compound to the cells, and the response was measured for 140 seconds. This is followed by second addition: 10 μL (5X) of an EC_{40} concentration of 5-HT agonist was added to the cells, and the response of the cells was measured for 125 seconds. Immediately, the third addition occurred by adding 12 mL assay buffer or 5X of an EC_{max} concentration of 5-HT and the response of the cells was measured for 90 seconds. DMSO vehicle in the first add was added to the control wells to ensure EC_{40} and EC_{max} responses. Calcium fluorescence was recorded as fold over basal fluorescence and raw data were normalized to the maximal response to 5-HT agonist. Compound-evoked increase in calcium response in the absence of 5-HT agonist was determined as agonist activity. Compound-evoked increase in calcium response in the presence of 5-HT EC_{40} agonist was determined as potentiator activity of positive allosteric modulator. Potency (EC_{50}) and maximum response (% 5-HT Max) for compounds was

determined using a four-parameter logistical equation using GraphPad Prism (La Jolla, CA) or the Dotmatics software platform (Woburn, MA):

$$y = bottom + \frac{top - bottom}{1 + 10^{(LogEC50 - A)Hillslope}}$$

where *A* is the molar concentration of the compound; *bottom* and *top* denote the lower and upper plateaus of the concentration-response curve; HillSlope is the Hill coefficient that describes the steepness of the curve; and EC_{50} is the molar concentration of compound required to generate a response halfway between the *top* and *bottom*.

5-HT$_{2B}$ and 5-HT$_{2C}$ Calcium Mobilization Assay. Functional activities of the compounds at human 5-HT$_{2B}$ and 5-HT$_{2C}$ were assessed by measuring the receptor-induced mobilization of intracellular calcium as described above. Chinese Hamster Ovary (CHO) cells stably expressing human 5-HT$_{2C}$ were cultured in F12 medium containing 10% dialyzed fetal bovine serum, 20 mM HEPES, 100 units/mL antibiotics/antimycotic, and 0.5 mg/ml G418. Plating density and assay conditions were identical to the 5-HT$_{2A}$ assay protocol described above. For the 5-HT$_{2B}$ cellular assay, tetracycline-inducible 5-HT$_{2B}$ Flp-In 293 T-Rex cells[2] were used. To induce 5-HT$_{2B}$ receptor expression, 2 μg/mL tetracycline was added when cells were seeded in amine-coated 384 cell plates (Corning) at 20,000 cells/20 μL density per well.

References

1. Melancon, B. J.; Hopkins, C. R.; Wood, M. R.; Emmitte, K. A.; Niswender, C. M.; Christopoulos, A.; Conn, P. J.; Lindsley, C. W. Allosteric Modulation of Seven Transmembrane Spanning Receptors: Theory, Practice, and Opportunities for Central Nervous System Drug Discovery. *J. Med. Chem*. **2012**, *55*, 1445-1464.

2. Kaplan, A. L.; Confair, D. N.; Kim, K.; Barros-Álvarez, X.; Rodriguez, R. M.; Yang, Y.; Kweon, O. S.; Che, T.; McCorvy, J. D.; Kamber, D. N.; Phelan, J. P.; Martins, L. C.; Pogorelov, V. M.; DiBerto, J. F.; Slocum, S. T.; Huang, X.-P.; Kumar, J. M.; Robertson, M. J.; Panova, O.; Seven, A. B.; Wetsel, A. Q.; Wetsel, W. C.; Irwin, J. J.; Skiniotis, G.; Schoichet, B. K.; Roth, B. L.; Ellman, J. A. Bespoke Library Docking for 5-HT$_{2A}$ Receptor Agonists with Antidepressant Activity. *Nature* **2022**, *610*, 582-591.

DMPK

IV PK and Plasma-Brain Level (PBL) Determination

All rodent PK experiments were conducted in accordance with the National Institute of Health regulations of animal care covered in Principles of Laboratory Animal Care (National Institutes of Health publication 85-23, revised 1985) and were approved by the Institutional Animal Care and Use Committee.

In-life phase

VU6067416 was formulated as a solution in 8% ethanol, 32% PEG400, and 60% DMSO (*v/v/v*) and administered as a single 0.2 mg/kg IV dose (0.5 mL/kg) to male, Sprague Dawley rats (*n* = 1; 342 gram body weights) via injection into a surgically-implanted jugular vein catheter. Blood samples were collected serially from a surgically implanted carotid artery catheter in each animal over multiple post-administration time points (0.033, 0.117, 0.25, 0.5, 1, 2, 4, 7, and 24 hours) into K_2EDTA anticoagulant-fortified tubes and immediately placed on wet ice. The blood samples were then centrifuged (1700 rcf, 5 minutes, 4 ˚C) to obtain plasma samples, which were stored at -80 ˚C until analysis by LC-MS/MS.

For determination of the brain over plasma ratio (K_p), VU6067416 was formulated in 8% ethanol, 32% PEG400 and 60% DMSO (*v/v/v*) and administered as a single 0.2 mg/kg IV dose (0.5 mL/kg) to male, Sprague Dawley rats (*n* = 1; 316 grams body weight) via injection into a surgically-implanted jugular vein catheter. At 15 min post dosing, a blood sample was collected from a surgically-implanted carotid catheter into a K_2EDTA anticoagulant-fortified tube and immediately placed on wet ice. The blood sample was then centrifuged (1700 rcf, 5 minutes, 4 ˚C) to obtain the plasma sample for this study. At the same post-dosing time point, the whole brain tissue sample was obtained by rapid dissection, rinsed with saline, and immediately frozen on dry ice in an individual tissue collection box (dry ice). All brain and plasma samples were stored at -80 ˚C until analysis by LC-MS/MS.

Sample preparation for bioanalysis

Plasma samples from the in-life phase of the study were thawed at ambient temperature (benchtop), and then aliquots (20 µL per sample) were transferred to a 96-shallow-well (V-bottom) plate. Matrix-matched quality control (QC) samples and a standard curve of VU6067416 (1 mg/mL DMSO stock solution) were prepared in blank rat plasma (K_2EDTA-treated) or blank brain homogenate via serial dilution and transferred (20 µL each) to the plate along with multiple blank plasma and brain homogenate samples. Acetonitrile (120 µL) containing IS (10 nM carbamazepine) was added to each well of the plate to precipitate protein. The plate was then centrifuged (4000 rcf, 5 minutes, ambient temperature), and resulting supernatants (60 µL each) were transferred to a new 96-shallow-well (V-bottom) plate containing an equal volume (60 µL per well) of water (Milli-Q purified). The plate was then sealed in preparation for LC-MS/MS analysis.

Preparation of brain samples was identical to that of plasma samples except for the following modifications: while thawing, brains were weighed (inside their collection boxes using a universal empty collection box tare weight) and then subjected to mechanical homogenization (Mini-BeadBeater™, BioSpec Products, Inc., Bartlesville, OK) in the presence of zirconia/silica beads (1.0 mm) and extraction buffer (isopropanol:water, 7:3, *v/v*; 3 mL per sample, corrected for post-quantitation). Homogenized brain samples were then centrifuged (4000 rcf, 5 minutes, ambient temperature), and 5 µL of the supernatant was diluted in 15 µL of blank plasma for quantification of the analyte. The plasma standard curve and QCs were used for VU6067416 quantitation in brain.

LC-MS/MS analysis

Prepared samples were injected (10 µL each) onto an AB Sciex Triple Quad 4500 mass spectrometer system with an Agilent 1260 Infinity II pump and autosampler. Mass spectrometer conditions are described in **Table 1**. Quantitation of VU6067416 was performed via AB Sciex Multiquant software using the raw analyte:IS peak area ratios. The typical detection range for VU6067416 was 0.5 ng/mL to ≥ 5,000 ng/mL utilizing a quadratic equation regression with $1/x^2$ weighting.

Correction for dilution of all brain samples (in extraction buffer and subsequently in blank plasma, as previously described) was performed post-quantitation. The corrections for dilution in extraction buffer employed correction factors specific to each brain weight.

Table S1. LC-MS/MS Conditions

Injection volume	10 µL	
Mobile phase A	0.5% Formic Acid in Water	
Mobile phase B	0.5% Formic Acid in Acetonitrile	
Flowrate	0.5 mL/min	
Gradient	Time	% Mobile Phase B
	0.0	5
	0.2	5
	0.8	95
	1.5	95
	1.7	5
	2.7	Stop
Column	Fortis C18 (50 x 3.0 mm, 3 µm)	
Data collection and analysis software/version	Analyst v. 1.7.1	
Ionization mode	Positive Electrospray	
Collision gas (psi)	9	
Curtain gas (psi)	40	
GS1 (psi)	40	
GS2 (psi)	40	
Capillary voltage (V)	5500	
Source TurboIonSpray® temp. (°C)	500	
MRM mass transitions (Da):<br>VU6067416<br>I.S.	 278.0/249.0 (CE: 19, DP: 67, EP: 10, CXP: 10)<br>237.0/193.9 (CE: 25, DP: 96, EP: 10, CXP: 8)	

Intrinsic Clearance

Determination of test compounds' *in vitro* intrinsic clearance (CL_{int}) and prediction of hepatic clearance (CL_{hep}) was performed via an in-house assay using human and/or rat hepatic microsomes with a substrate depletion approach according to previously described methodology.[1-8]

Plasma Protein Binding

Determination of VU6067416 fraction unbound (f_u) in plasma from mouse, rat and human was conducted *in vitro* via equilibrium dialysis using HTDialysis membrane plates. The top half of the plate was filled with 100 µL of Dulbecco's Phosphate Buffered Saline, pH 7.4 (DPBS). VU6067416 was diluted into plasma from each species (5 µM final concentration), which was aliquoted in triplicate to the 'bottom half' of the prepared HTD plate wells. The HTD plate was sealed and incubated for 6 hours at 37 ˚C. Following incubation, each well (both top and bottom halves) were transferred (20 µL) to the corresponding wells of a 96-shallow-well (V-bottom) plate. The daughter plates were then matrix-matched (DPBS side wells received equal volume of plasma, and plasma side wells received equal volume of DPBS), and extraction solution (120 µL; acetonitrile containing 50 nM carbamazepine as IS) was added to all wells of both daughter plates to precipitate protein and extract test article. The plates were then sealed and centrifuged (3500 rcf) for 10 minutes at ambient temperature. Supernatant (60 µL) from each well of the daughter plates was then transferred to the corresponding wells of new daughter plates (96-shallow-well, V bottom) containing water (Milli-Q, 60 µL/well), and the plates were sealed in preparation for LC-MS/MS analysis (see LC-MS/MS analysis method above).

The unbound fraction (f_u) was calculated following the equation below, and mean values for each species were calculated from 3 replicates.

$$\frac{\text{mean DPBS well ratio}}{\text{mean plasma well ratio}}$$

P-gp Effflux

Determination of test compounds' potential for efflux by human P-gp at a single concentration (5 µM; in duplicate) was performed via contract by Pharmaron (Exton, PA) using a bidirectional permeability assay with MDCK-MDR1 cells in accordance with their established standard assay methodology.

References

1. Obach, R. S. Prediction of Human Clearance of Twenty-Nine Drugs from Hepatic Microsomal Intrinsic Clearance Data: An Examination of In Vitro Half-Life Approach and Nonspecific Binding to Microsomes. *Drug Metab. Dispos*. **1999**, *27*, 1350-1359.

2. Houston, J. B. Utility of In Vitro Drug Metabolism Data In Predicting In Vivo Metabolic Clearance. *Biochem. Pharmacol*. **1994**, *47*, 1469-1479.

3. Lin, J. H.; Chiba, M.; Balani, S. K.; Chen, I .W.; Kwei, G. Y.; Vastag, K. J.; Nishime, J. A. Species Differences in the Pharmacokinetics and Metabolism of Indinavir, a Potent Human Immunodeficiency Virus Protease Inhibitor. *Drug. Metab. Dispos*. **1996**, *24*, 1111-1120.

For additional examples from our laboratory, see:

4. Wenthur, C. J.; Morrison, R.; Felts, A. S.; Smith, K. A.; Engers, J. L.; Byers, F. W.; Daniels, J. S.; Emmitte, K. A.; Conn, P. J.; Lindsley, C. W. Discovery of (R)-(2-Fluoro-4-((-4-methoxyphenyl)ethynyl)phenyl) (3-Hydroxypiperidin-1-yl)methanone (ML337), An mGlu3 Selective and CNS Penetrant Negative Allosteric Modulator (NAM). *J. Med. Chem*. **2013**, *56*, 5208-5212.

5. Bridges, T. M.; Rook, J. M.; Noetzel, M. J.; Morrison, R. D.; Zhou, Y.; Gogliotti, R. D.; Vinson, P. N.; Xiang, Z.; Jones, C. K.; Niswender, C. M.; Lindsley, C. W.; Stauffer, S. R.; Conn, J. P.; Daniels, J. S. Biotransformation of a Novel Positive Allosteric Modulator of Metabotropic Glutamate Receptor Subtype 5 Contributes to Seizure-Like Adverse Events in Rats Involving a Receptor Agonism-Dependent Mechanism. *Drug. Metab. Dispos*. **2013**, *41*, 1703-1714.

6. Engers, D. W.; Blobaum, A. L.; Gogliotti, R. D.; Cheung, Y.-Y.; Salovich, J. M.; GarciaBarrantes, P. M.; Daniels, J. S.; Morrison, R.; Jones, C. K.; Soars, M. G.; Zhuo, X.; Hurley, J.; Macor, J. E.; Bronson, J. J.; Conn, P. J.; Lindsley, C. W.; Niswender, C. M.; Hopkins, C. R. Discovery, Synthesis, and Preclinical Characterization of N-(3-Chloro-4- fluorophenyl)-1H-pyrazolo[4,3-b]pyridin-3-amine (VU0418506), a Novel Positive Allosteric Modulator of the Metabotropic Glutamate Receptor 4 (mGlu4). *ACS Chem. Neurosci.* **2016**, *7*, 1192-1200.

7. Reed, C. W.; Yohn, S. E.; Washecheck, J. P.; Roenfanz, H. F.; Quitalig, M. C.; Luscombe, V. B.; Jenkins, M. T.; Rodriguez, A. L.; Engers, D. W.; Blobaum, A. L.; Conn, P. J.; Niswender, C. M.; Lindsley, C. W. Discovery of an Orally Bioavailable and Central Nervous System (CNS) Penetrant mGlu7 Negative Allosteric Modulator (NAM) in Vivo Tool Compound: N-(2-(1H-1,2,4-triazol-1-yl)-5-(trifluoromethoxy)phenyl)-4- (cyclopropylmethoxy)-3-methoxybenzamide (VU6012962). *J. Med. Chem.* **2019**, *62*, 1690-1695.

8. Spock, M.; Carter, T. R.; Bollinger, K. A.; Han, C.; Baker, L. A.; Rodriguez, A. L.; Peng, L.; Dickerson, J. W.; Qi, A.; Rook. J. M.; O'Neill, J. C.; Watson, K. J.; Chang, S.; Bridges, T. M.; Engers, J. L.; Engers, D. W.; Niswender, C. M.; Conn, P. J.; Lindsley, C. W.; Bender, A. M. Discovery of VU6028418: A Highly Selective and Orally Bioavailable M_4 Muscarinic Acetylcholine Receptor Antagonist. *ACS Med. Chem. Lett*. **2021**, *12,* 1342-1349.

Docking Experiments

The cryo electron microscopy structures of a complex containing the active state of 5-HT_{2A}R at a resolution of 3.45Å (PDB ID: 7RAN)[1,2] and a complex containing the active state of 5-HT_{2B}R at a resolution of 2.90Å (PDB ID: 7SRR)[3,4] were retrieved from the Protein Data Bank.[5] The protein complex was visualized using ChimeraX,[6] and the extraneous protein chains were removed (e.g. mini-Gq, scFv16), along with the bound agonist. Hydrogens were added with optimization for intramolecular hydrogen bond formation. The protein was further prepared using AutoDock Tools,[7] wherein all nonpolar hydrogens were merged and Gasteiger charges were added.

Compound **19d** was constructed in ChemDraw, then its protonation state was assigned according to physiological pH. The structure was imported into Chem3D for preliminary MM2 minimization, then prepared for docking by merging nonpolar hydrogens and adding Gasteiger charges using AutoDock Tools.[7] The prepared structure was then docked using Autodock VinaXB[8] to the prepared 5-HT_{2A} receptor according to the following parameters: Grid Box: 40x46x48; Exhaustiveness: 20; Conformers: 20; Energy Cutoff: 1.9 kcal/mol; and to the prepared 5-HT_{2B} receptor according to the following parameters: Grid Box: 34x44x36; Exhaustiveness: 20; Conformers: 20; Energy Cutoff: 3.0 kcal/mol. Results were aggregated and analyzed based on a combination of docking score and visual inspection of the binding mode. Visualization was conducted with ChimeraX.[6] Halogen bond formation was assessed based on literature cutoffs for halogen bond formation: C-Br···O angle >140°, Br···O distance < 3.37 Å.[9]

UCSF ChimeraX was developed by the Resource for Biocomputing, Visualization, and Informatics at the University of California, San Francisco, with support from National Institutes of Health R01-GM129325 and the Office of Cyber Infrastructure and Computational Biology, National Institute of Allergy and Infectious Diseases.

References

1. Kaplan, A. L.; Confair, D. N.; Kim, K.; Barros-Álvarez, X.; Rodriguiz, R. M.; Yang, Y.; Kweon, O. S.; Che, T.; McCorvy, J. D.; Kamber, D. N.; Phelan, J. P.; Martins, L. C.; Pogorelov, V. M.; DiBerto, J. F.; Slocum, S. T.; Huang, X.-P.; Kumar, J. M.; Robertson, M. J.; Panova, O.; Seven, A. B.; Wetsel, A. Q.; Wetsel, W. C.; Irwin, J. J.; Skiniotis, G.; Shoichet, B. K.; Roth, B. L.; Ellman, J. A. Bespoke Library Docking for 5-HT_{2A} Receptor Agonists with Antidepressant Activity. *Nature* **2022**, 582–591.

2. Barros-Alvarez, X.; Kim, K.; Panova, O.; Roth, B. L.; Skiniotis, G. 5-HT2AR bound to a novel agonist in complex with a mini-Gq protein and an active-state stabilizing single-chain variable fragment (scFv16) obtained by cryo-electron microscopy (cryoEM). https://doi.org/10.2210/pdb7RAN/pdb.

3. Cao, C.; Barros-Álvarez, X.; Zhang, S.; Kim, K.; Dämgen, M. A.; Panova, O.; Suomivuori, C.-M.; Fay, J. F.; Zhong, X.; Krumm, B. E.; Gumpper, R. H.; Seven, A. B.; Robertson, M. J.; Krogan, N. J.; Hüttenhain, R.; Nichols, D. E.; Dror, R. O.; Skiniotis, G.; Roth, B. L. Signaling Snapshots of a Serotonin Receptor Activated by the Prototypical Psychedelic LSD. *Neuron* **2022**, *110*, 3154-3167.

4. Barros-Alvarez, X.; Kim, K.; Panova, O.; Cao, Can; Roth, Bryan L.; Skiniotis, Georgios. 5-HT_{2B} receptor bound to LSD in complex with heterotrimeric mini-Gq protein obtained by cryo-electron microscopy (cryoEM). https://doi.org/10.2210/pdb7SRR/pdb.

5. Berman, H. M. The Protein Data Bank. *Nucleic Acids Res*. **2000**, *28*, 235–242.

6. Meng, E. C.; Goddard, T. D.; Pettersen, E. F.; Couch, G. S.; Pearson, Z. J.; Morris, J. H.; Ferrin, T. E. UCSF ChimeraX : Tools for Structure Building and Analysis. *Protein Sci.* **2023**, *32*, e4792.

7. Sanner, M. F. Python: A Programming Language for Software Integration and Development. *J. Mol. Graph. Model*. **1999**, *17*, 57–61.

8. Koebel, M. R.; Schmadeke, G.; Posner, R. G.; Sirimulla, S. AutoDock VinaXB: Implementation of XBSF, New Empirical Halogen Bond Scoring Function, into AutoDock Vina. *J. Cheminformatics* **2016**, *8*, 27.

9. Xu, Z.; Yang, Z.; Liu, Y.; Lu, Y.; Chen, K.; Zhu, W. Halogen Bond: Its Role beyond Drug–Target Binding Affinity for Drug Discovery and Development. *J. Chem. Inf. Model*. **2014**, *54*, 69–78.
